# Rapid Morphological Change in the Masticatory Structures of an Important Ecosystem Service Provider

**DOI:** 10.1371/journal.pone.0127218

**Published:** 2015-06-10

**Authors:** John W. Doudna, Brent J. Danielson

**Affiliations:** Ecology, Evolution, and Organismal Biology Department, Iowa State University, Ames, Iowa, United States of America; Team 'Evo-Devo of Vertebrate Dentition', FRANCE

## Abstract

Humans have altered the biotic and abiotic environmental conditions of most organisms. In some cases, such as intensive agriculture, an organism’s entire ecosystem is converted to novel conditions. Thus, it is striking that some species continue to thrive under such conditions. The prairie deer mouse (*Peromyscus maniculatus bairdii*) is an example of such an organism, and so we sought to understand what role evolutionary adaptation played in the success of this species, with particular interest in adaptations to novel foods. In order to understand the evolutionary history of this species’ masticatory structures, we examined the maxilla, zygomatic plate, and mandible of historic specimens collected prior to 1910 to specimens collected in 2012 and 2013. We found that mandibles, zygomatic plates, and maxilla have all changed significantly since 1910, and that morphological development has shifted significantly. We present compelling evidence that these differences are due to natural selection as a response to a novel and ubiquitous food source, waste grain (corn, *Zea mays* and soybean, *Glycine max*).

## Introduction

Rapid changes to environmental conditions such as climate and landscape have become the normal conditions under which contemporary species must survive and reproduce. Therefore, rapid microevolutionary changes may be critical to the survival of species in an anthropogenic world. Rates of morphological adaptation of vertebrates, once thought to be incommensurate with ecological time scales, transpire quickly in some species under novel ecosystem pressures [1,2,3,4,5,6]. Surprisingly, few studies have examined morphological changes due to landscape change [7, 8]. Intensive agriculture provides a natural experiment of rapid and extensive modifications to food and habitats in ways that are almost certain to represent strong natural selection. This rapid change provides an opportunity to study the rate at which a species can change to cope with a dramatically new environment.

*Peromyscus maniculatus bairdii* is an example of a species that has experienced drastic shifts in its environmental conditions over a short time period. *P*.*m*. *bairdii* is the prairie form of the common deer mouse, and are constrained to grassland landscapes throughout the Midwestern USA (Midwest), where humans have converted more than 80% of the prairie habitat to agriculture [9]. Within this region, corn-soybean agriculture dominates the eastern and central regions, while the western, southern and northern edges of the Midwest has a lower percentage of corn-soybean, with other land cover types that lack large seeded crops [10].

The shift from a prairie landscape to row crop was a rather dramatic one in the Midwest. For example, in Iowa in 1860, there were only about 800,000 ha of row crops, but there were 4 million ha by 1910 [11]. Thus, the period between 1860 and 1910 was characterized by local and regionally mixed cover types. More broadly, in 1926, 40 million ha of corn, 800,000 ha of soybean, and 18 million ha of oats were planted in the United States [10]. The amount of soybean increased to 16 million ha by 1967 while corn declined to 29 million ha, and oats declined to 8 million ha as soybean became a common rotation crop with corn and fewer farm animals needed oats for feed [10]. Thus, *P*. *m*. *bairdii* experienced a change in the landscape between 1860 and 1910 which led to a much more homogenized landscape, both locally and regionally. This trend continued until mid-century, when the amount of corn and soybean in the landscape stabilized [10]. Thus, 1910 marks the beginning of a period when natural habitat was extremely limited and row crops dominated the landscape in vast regions of the Midwest. This date also marks the beginning of row crop agriculture as the ubiquitous selective environment in the Midwest and the point when *P*. *m*. *bairdii* likely became the most common resident vertebrate of Midwestern agriculture [12].

Part of the explanation for the success of *P*.*m*. *bairdii* in this new system may be the species’ tolerance of a novel diet of native and non-native insects and non-native weed seeds and a preference for crop seeds that are left after harvest [12,13,14,15]. While the non-native insects and weed seeds may be analogous to native species, corn and soybean are completely novel food items introduced by humans. In addition, the large quantity of this high-quality waste grain makes it the most important winter source of calories in agricultural fields [16,17]. Dried corn and soybean are hard seeds that are more than an order of magnitude larger than other common prairie seeds (corn: 0.3 g; soybean: 0.12 g; wild sunflower: 0.007g [18]), and this size difference may exert significant selective pressure on masticatory structure morphology.

In fact, we have found that the mice strongly prefer waste grain (corn and soybean) relative to any other seed type we tested (unpublished data). In order for the mice to process these relatively large but nutritious seeds, they may benefit from a new masticatory morphology specialized for the task of processing the large, hard seeds. We also know that jaw gape and bite force are related, and that the morphology of the skull can be an important factor in the relationship of these two parameters [19,20,21]. Due to this relationship, we expect that mandible morphology may be strongly correlated to feeding efficiency in the species, and that waste grain may represent a significant selective pressure on morphology.

Previous studies of dietary effect on morphology have found that diet can induce plastic responses in masticatory structures, especially the condyle, due to food hardness [22]. The same study found that contemporary evolution (*sensu* [23]) can result from a significant change in food hardness, especially in muscle attachment regions. *Peromyscus maniculatus bairdii* could be an example of such contemporary evolution. In order to determine the role of human-modified landscapes in this species’ evolution, we predict that, since 1910, the zygomatic plate and ramus region would increase in size relative to the whole specimen because they would increase the attachment area for the masseter muscle complex. We also predict that the coronoid process in contemporary specimens would be larger to provide greater muscle attachment area for the temporalis muscle complex. Additionally, we predict that the condyle of the mandible would be larger due to the hardness of new foods, as seen in a previous diet study [22]. Finally, we predict that variability in corn-soybean production intensity would be predictive of morphological changes.

## Methods

### Digitization

**Fig 1:**
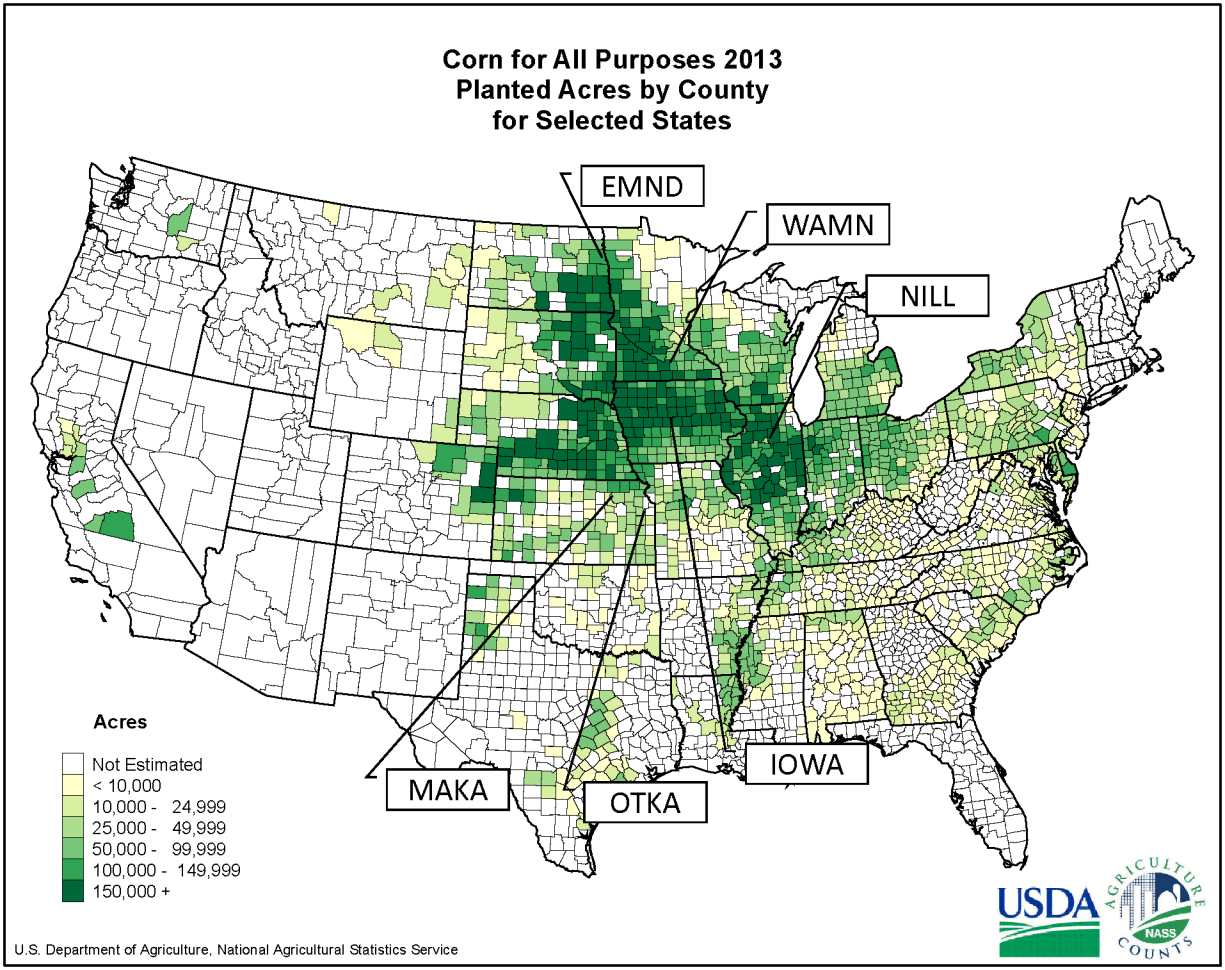
Locations of *Peromyscus maniculatus bairdii* trapping. The map illustrates the spatial distribution of corn-soybean agricultural intensity in the Midwestern US. The locations indicated on the map are detailed in Table 1. They were chosen because concentrations of historical specimens from before 1910 were available from the region. Three of our locations (NILL, IOWA, and WAMN) are in regions of greater than 75% corn-soybean agriculture cover within the county, while the other three (MAKA, OTKA, and EMND) are in agricultural regions of less than 33% corn and soybean cover within the county. All contemporary specimens were collected from corn-soybean fields. Map available at: http://www.nass.usda.gov/Charts_and_Maps/Crops_County/cr-pl.asp.

In order to test the hypothesis that mice have evolved to have stronger jaw morphologies, historic (1870–1910) and contemporary (2012–2013) specimens of *Peromyscus maniculatus bairdii* were compared. A collection of historic specimens was created, starting with a search of the online Global Biodiversity Information Facility database for museum collections of *P*. *m*. *bairdii* skulls from before 1910 [24]. In order to generate direct comparisons of historic and contemporary species, the possible specimens were narrowed based on regional concentrations of historical specimens. This research found concentrated collections in northeastern Illinois, central and south-central Iowa, northeastern Kansas (2 locations), southwestern Minnesota, and northeastern North Dakota (Fig 1 and Table 1). Specimens were shipped from the National Museum of Natural History (Washington, D.C.), and we visited the Field Museum of Natural History (Chicago, IL), University of Kansas Biodiversity Research Center (Lawrence, KS), and University of Iowa Museum of Natural History (Iowa City, IA).

**Table 1:** Summary of specimen collection sites.

	Location		% Corn & Soybean 2012
	Before 1910	2012–2013	
Illinois (NILL)	Cook and Lake County (N = 16)	41.83913,-88.865365 (N = 24)	81
Iowa (IOWA)	Knoxville and Central Iowa (N = 25)	41.990805, -93.685187 (N = 28)	76
Minnesota (WAMN)	Fort Snelling (N = 15)	44.070972,-93.525711 (N = 26)	75
North Dakota (EMND)	Northeast North Dakota (N = 22)	47.953432,-97.434925 (N = 31)	33
Ottawa, KS (OTKA)	Lawrence (N = 40)	38.537739,-95.245275 (N = 35)	21
Manhattan, KS (MAKA)	Onaga (N = 32)	39.213044,-96.595392 (N = 16)	14

Historical location is an estimate from museum records and contemporary locations are the coordinates of the sampled field. Percent corn and soybean in 2012 is based on National Agricultural Statistics Service numbers (nass.usda.gov). The number of acres of land planted to corn or soybean was divided by the recorded size of each county in which sampling occurred.

Based on historic specimens, six concentrations of specimens were identified that would allow for direct comparison to contemporary specimens. Contemporary specimens were collected from each of the locations identified from this process. Mice were snap trapped, using Museum Specials (Woodstream Corporation, Lititz, Pennsylvania) within corn and soybean fields at each location, following recommendations in the Guide for the Care and Use of Laboratory Animals of the National Institutes of Health. The protocol was approved by the Institutional Animal Care and Use Committee of Iowa State University (Permit Number: 4-12-7335-W). In parallel with the historical specimens, specimens were collected from six locations: Ames IA, Ottawa KS, Manhattan KS, Emerado ND, Waseca MN, and Shabbona IL (Fig 1 and Table 1). The Iowa, Minnesota, and Illinois locations are in counties with greater than 75% corn-soybean landcover while the two Kansas locations and the North Dakota location were in a county with less than 33% corn-soybean landcover in 2013 (Table 1). At each location, up to 40 adult *P*. *m*. *bairdii* were collected. Thus, snap traps were set for 1–3 nights (checking traps each morning), depending on trap efficiency at each location, between July and September 2012 in Iowa, Kansas, North Dakota, and Minnesota. Traps were set for 1–3 nights in July 2013 again in Minnesota and in Illinois. Skulls were removed and placed in a dermestid beetle colony until clean.

**Fig 2:**
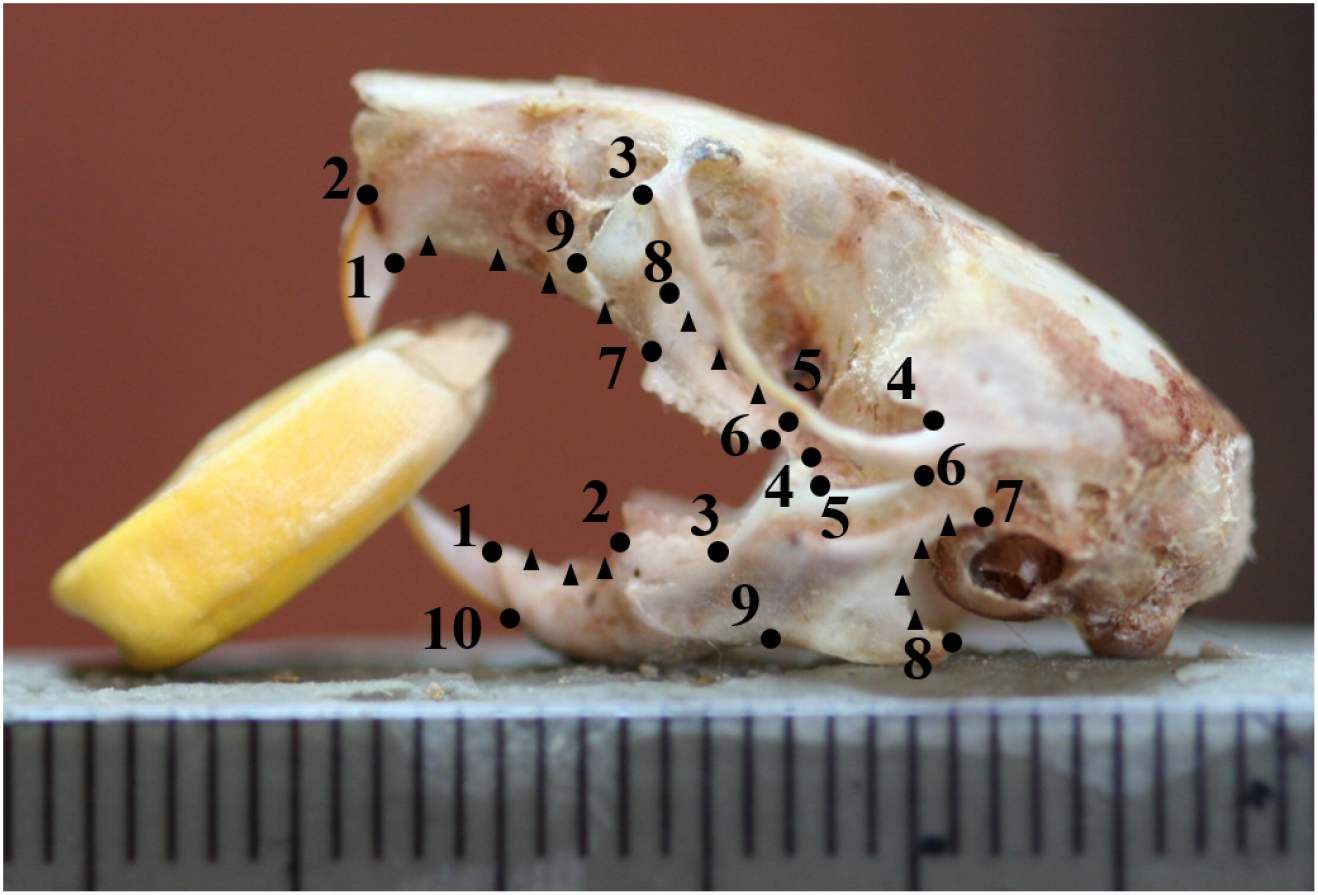
Landmarks (circles) and semilandmarks (triangles) digitized onto each specimen. Landmarks are detailed in Table 2.

In order to determine the changes in shape of *P*. *m*. *bairdii* jaws, data were collected from upper and lower jaw structures associated with masticatory muscle attachments. To this end, photographs were taken of the left, lateral perspective of skulls and mandibles separately for all specimens. A few exceptions occurred when historic specimens could not be disarticulated, so photographs were taken with skulls and mandibles attached. Occasionally, photographs of the right side of the specimen were taken, when the left side was too damaged for analysis. A setup of a digital camera with a macro lens was used, set at approximately 0.5m from the specimen, and a mm ruler was oriented along the long axis of the specimen and camera lens. Jaws were aligned so that the left side of the left mandible was aligned with the ruler, while the skull was aligned so that the left zygomatic arch was aligned with the ruler, and the sagittal suture was parallel to the ruler. A Canon EOS XT with an 8.0 MP sensor with a 100mm macro lens (EF = 1:2.8) was used for all photography. After all pictures had been collected, upper and lower jaw landmarks were digitized for all specimens. Landmarks followed McPhee and Myers et al. [25,26], but were modified for this species and question (Fig 2 and Table 2). We chose to exclude incisor and individual tooth landmarks due to tooth wear and movement or loss. Nine landmarks and 7 semilandmarks were digitized on the upper jaw, and 10 landmarks and 7 semilandmarks were digitized on the mandible. Landmarks were a combination of Type 1 and Type 2 landmarks (points of intersection of structures and points of maximum or minimum curvature). Semilandmarks are landmarks along a curve that are moved along that curve during the analysis. All specimens were digitized in tpsDig2 ([27], vers. 2.17). In order to test for errors associated with order of photography, a random subset of 25% of the specimens were re-photographed. No evidence was found that specimens from the original and follow-up test were different (Mean Square Error [MS] = 0.00247, p = 0.22). A random subset of 10% of specimens were re-digitized and a significant effect of practice time was found on digitization of upper jaws, but not mandibles. Therefore, all upper jaws were re-digitized in random order to remove experience bias.

**Table 2:** Landmarks used for morphological analysis.

Landmark	Mandibles	Upper Jaws
**1**	Dorsal insertion of incisor	Posterior insertion of incisor
**2**	Anterior of molar row	Anterior insertion of incisor
**3**	Posterior of visible molar row	Point of maximum curvature
**4**	Point of maximum curvature of coronoid process (temporalis attachment)	Point of maximum curvature of squamosal bone—posterior of zygomatic arch
**5**	Point of maximum curvature at base of coronoid process	Dorsal of maxillary-basisphenoid suture
**6**	Dorsal point of condylar head	Anterior end of molar row
**7**	Ventral point of condylar head	Posterior end of molar row
**8**	Point of maximum curvature	Point of maximum curvature
**9**	Point of maximum curvature (masseter attachment)	Point of maximum curvature
**10**	Ventral insertion of incisor	

Landmarks follow Myers et al. and McPhee [26,25]. Landmarks follow the same numbering as seen in Fig 2.

### Statistical Methods

Modern geometric morphometric analyses were conducted separately on mandibles and upper jaws using *geomorph* in the R statistical environment (vers. 3.0.2) [28,29]. These analyses use a generalized Procrustes analysis (GPA: [30,31]) to remove variation in rotation and position of digitized specimens. The centroid size (Csize) of each specimen is determined, and specimens are scaled to a Csize of 1 for all further processing. The GPA process leaves only metrics of shape in the scaled, rotated, and aligned coordinates [32]. During the GPA process, semilandmarks are also slid along a curve that minimizes bending energy and differences among specimens [33]. Following these steps, all statistical analyses are conducted on the rotated, scaled, and aligned landmarks of all specimens (unless otherwise noted). A common allometric component (CAC), a standardized measure of specimen shape relative to the mean specimen shape, was also calculated [34].

Based on the Csizes generated above, static allometry, a change in shape correlated with growth in size but not through developmental stages, was examined. Multivariate shape matrices were regressed on Csize to determine if there is a significant trend in shape change with size. This analysis produces Procrustes distances, and the sum of squares is evaluated against a Procrustes ANOVA, which performs similarly to a permutational MANOVA and was permuted 999 times in this study [35,36]. To test for different slopes in allometry, Csize was treated as a covariate, while time period was tested as a factor predicting the CAC using ANCOVA [37]. Visual inspection of the regression residuals revealed no patterns.

In order to determine if significant shape differences exist among groups, the effects of period (before 1910 or 2012–2013), geographic location, centroid size, and interactions were assessed as predictors of morphology, using Procrustes distance as described above. Following this, thin plate spline deformation grids qualitatively described the changes in shape between historic (before 1910) and contemporary (2012–2013) specimens by representing the difference between the mean of the historical and contemporary specimens. This is accomplished by determining the minimum bending energy required to change one shape into the other [38]. In this presentation, two structures that are identical produce a grid of equal-sized cells with horizontal and vertical lines perpendicular [38]. If there are differences in the relative size of a portion of a structure, this is illustrated as differences in the length between lines. A change in the shape of a region is illustrated by non-parallel and non-straight lines.

Following the qualitative depiction of change, we tested whether the changes seen represent increased biomechanical strength [39]. This assessment of biomechanical bite strength compares the ratios of the length for two muscle attachment areas to two lengths of leverage. Since biomechanical ratios represent a measure of bite strength, larger values represent increased bite force. The leverage values are the length from the jaw pivot point to the incisor and from the pivot to the molar. In our study, due to issues of differential tooth wear and stability, we used the insertion point of the tooth in the jaw (landmark 1) as the end of these two lengths. The muscle attachments for the masseter and temporalis muscle spanned from the condyle tip (landmark 6) to the ventral point of maximum curvature (landmark 9) and coronoid process tip (landmark 4), respectively. We then used ANOVA to evaluate whether ratios were significantly larger for contemporary specimens than historical specimens, which would indicate an increase in biomechanical strength.

In sum, we analyzed 150 historical and 160 contemporary specimens (Table 1). The first two axes of upper jaws and mandibles explained 38 and 40 percent of the variation in the data sets. Greater than 95% of the variation was explained within the first 17 axes of both datasets (out of 32 and 34 axes).

## Results

**Fig 3:**
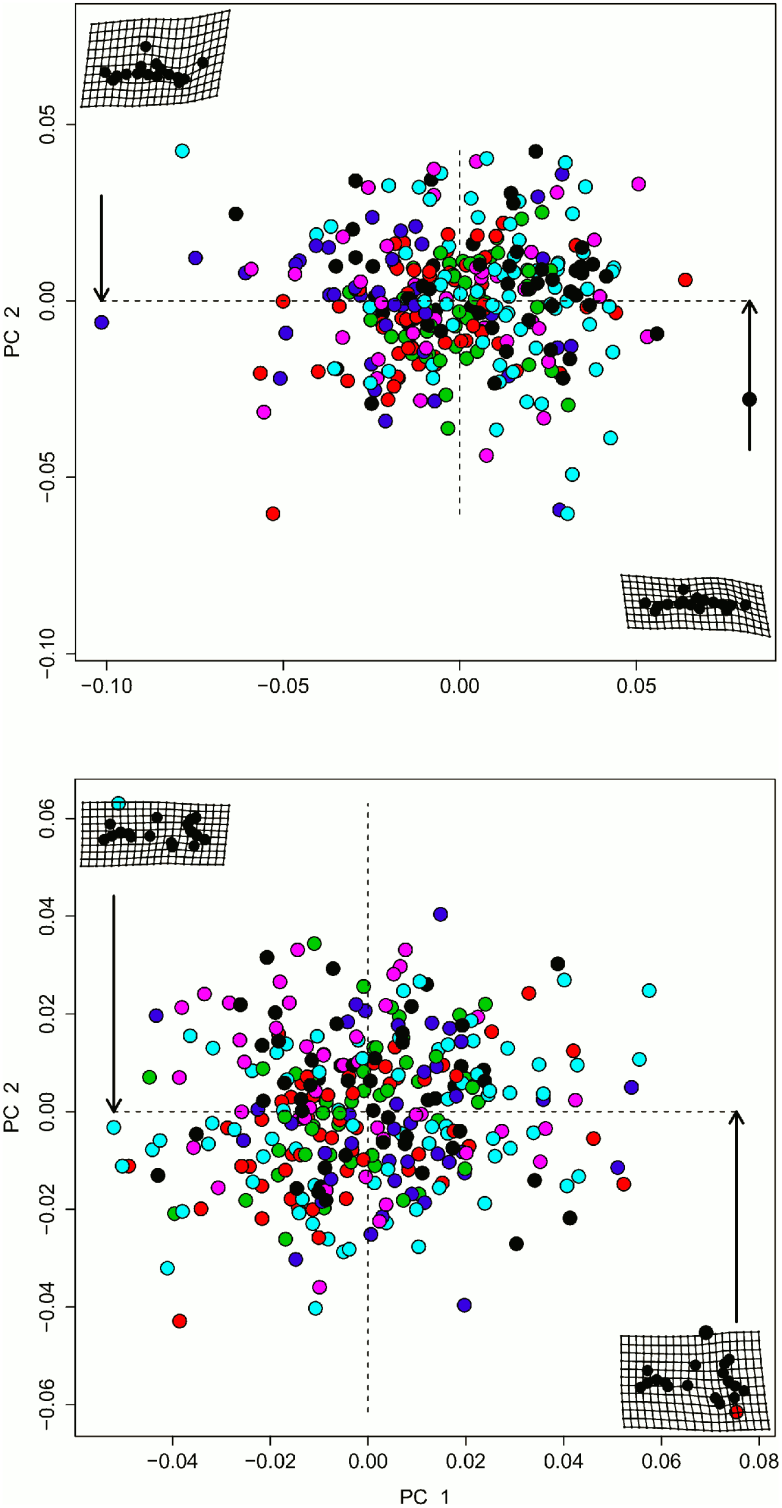
Tangent space for upper jaws (top) and mandibles (bottom). PC axes 1 and 2 explain the maximum amount of variation in the data (upper jaws: 27 and 13%, mandibles: 25 and 13%). The thin plate spline deformation grids in each corner represent the largest difference among all specimens, by indicating what each structure looks like at the ends of PC axis 1 relative to a specimen at the origin (0,0), as described in the methods. Colors are locations as follows: Black: EMND; Red: IOWA; Green: MAKA; Blue: NILL; White: OTKA; Grey: WAMN. Sample sizes and abbreviations are as in Table 1.

**Fig 4:**
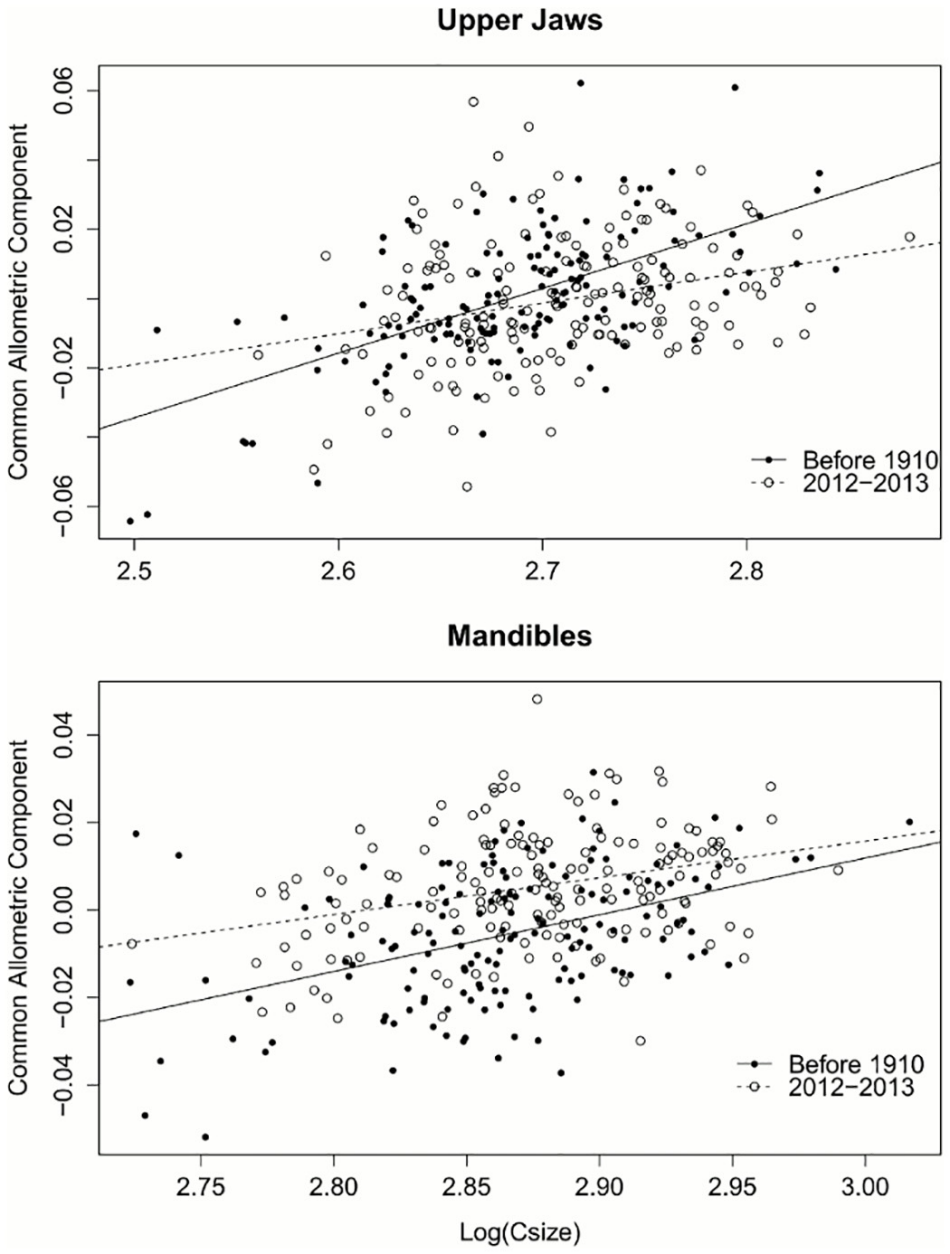
Allometry of upper jaws and mandibles by time period. Prediction lines represent a regression of shape values (common allometric component) within time period on the log of the centroid size [Log(Csize)], based on 150 historic and 160 contemporary specimens. Allometry tests show significant effects of size and year on shape for both structures. Interaction of size:period is significant for upper jaws, but not mandibles. Regression lines are historic upper jaws: CAC = 0.1876*Csize-0.5037; contemporary upper jaws: CAC = 0.08492*log(Csize)- 0.23; historic mandibles: CAC = 0.134*log(Csize)– 0.3886; contemporary mandibles: CAC = 0.1005*log(Csize)– 0.284.

Along PC axis 1, both the upper jaw and mandible are dorsoventrally wider (Fig 3 and Table 3). Mouse upper jaw and mandible morphology are significantly different between historical and contemporary specimens across the Midwestern US and within each location (Table 4). We also found significant static allometry of size and shape for jaws across all specimens, as evidenced by size-shape relationships within the same developmental category (all of our specimens were adults) (Table 4) (Fig 4). In addition to size, period is a significant predictor of the allometric regression intercepts after accounting for location (upper jaw: -0.23 vs. -0.50, F_1,305_ = 3.96, p = 0.048; mandibles: -0.28 vs. -0.39, F_1,305_ = 42.25, p < 0.001; Fig 4). Further investigation of these patterns revealed that allometric slopes (interactions of period and size) of contemporary specimens were shallower than historic specimens (0.08 vs. 0.19 and 0.09 vs. 0.13; Fig 4), with a significant effect of period on slope for upper jaws (MSE = 0.003, F_1,305_ = 10.48, p = 0.001). In contrast, slopes of allometry did not interact with time period for mandibles, suggesting a consistent difference due to time period for all sizes (MSE = 0.0004, F_1,305_ = 2.14, p = 0.14). In total, contemporary and historic upper jaws grew along different shape trajectories (illustrated by different and intersecting slopes), while mandibles grew along the same trajectory of shape change, but through different regions of morphospace (illustrated by non-intersecting slopes of different elevations).

**Table 3:** Summary of variation explained by PC axes.

Axes	Mandibles	Upper Jaws
	%	%
**1**	25	27
**2**	13	13
**3**	11	9
**4**	10	8
**5**	7	6
**6**	5	5
**7**	5	4
**8**	3	4

This table shows the percent of variation explained by the first 8 PC axes of mandible and upper jaw shape.

**Table 4:** Summary of predictors of jaw morphology.

Mandibles	
Parameters	MS Value
**Location**	0.004***
**Period**	0.027***
**Location x Period**	0.005***
**Size**	0.013 ***
**Period x Size**	0.0019 (p = 0.44)
**Upper Jaws**	
**Parameters**	MS Value
**Location**	0.009***
**Period**	0.011**
**Location x Period**	0.009***
**Size**	0.018 ***
**Period x Size**	0.008 **

Location, period, size, and interactions were modeled as predictors of mandible and upper jaw morphology, using a Procrustes distance metric and resampling techniques. Values are mean square errors explained by each parameter, based on all 310 specimens as described in Table 1. Significance codes are as follows:

*** p < 0.001,

** p < 0.01,

* p < 0.05,. p < 0.10.

**Fig 5:**
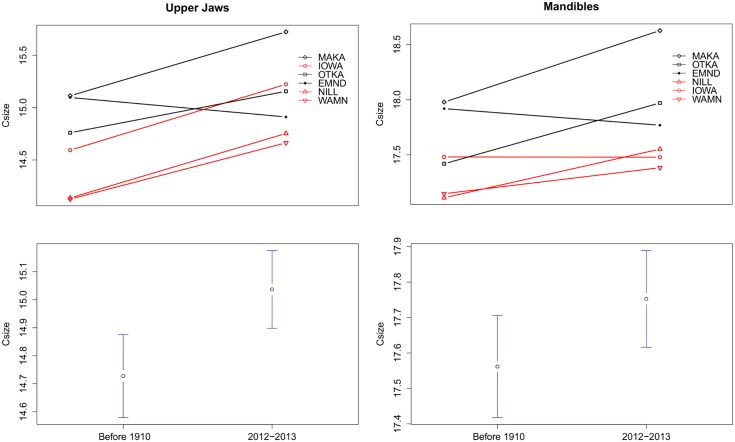
Size differences in mouse upper jaws and mandibles. All data depict average centroid size, which is calculated using all landmarks included in the morphometric analysis. Figures depict mandible and upper jaw size differences by location and year. Site abbreviations and sample sizes are as in Table 1. Sites with greater than or equal to 75% corn-soybean cover in the landscape are depicted in red, while sites with less than or equal to 33% corn-soybean cover are depicted in black. Bottom graphs illustrate the average size and 1 SE by time period.

Not only did time periods have different allometric growth patterns, contemporary masticatory structures are also significantly larger than historic structures. Upper jaw size increased significantly from 14.73 mm (0.92, 1SD) to 15.03 mm (0.89) (MSE = 7.42, F_1,299_ = 10.15, p = 0.002). The average size of mandibles also increased significantly from 17.56 mm (0.89) to 17.74 mm (0.87) (MSE = 2.825, F_1,299_ = 4.10, p = 0.04) (Fig 5). Location was also a significant predictor of Csize for upper jaws and mandibles, but there were no significant interactions between the predictors (location: MSE = 5.63, F_5,299_ = 7.70, p < 0.001 and MSE = 5.84, F_5,299_ = 8.48, p < 0.001; interaction effects: p = 0.13 and 0.09) (Fig 5).

**Fig 6:**
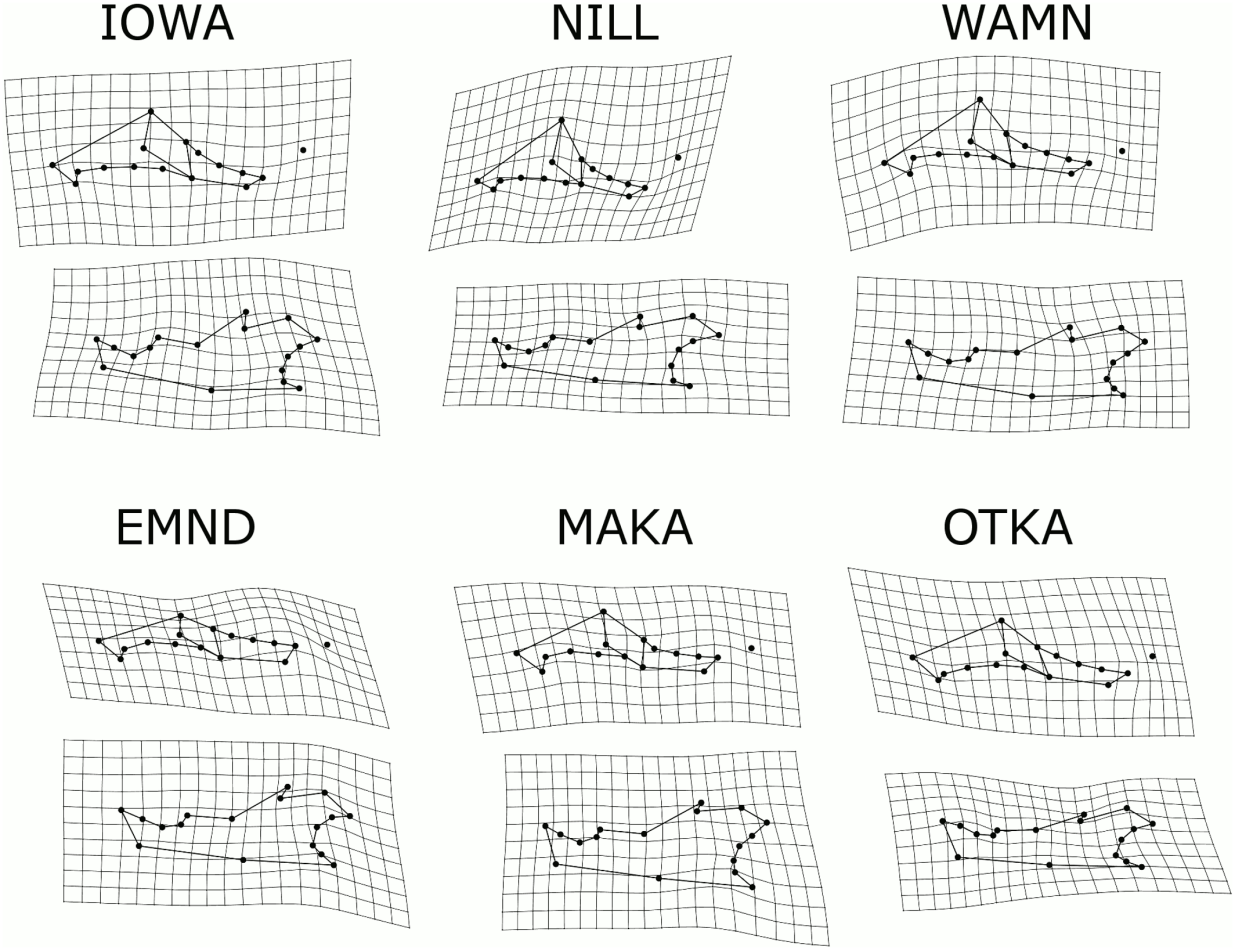
Location-specific changes in *Peromyscus maniculatus bairdii* jaw shape. Thin plate spline (TPS) deformation grids illustrate the changes in shape that have occurred in each location from approximately 1910 to 2012. TPS grids represent a hypothetical change in shape, based on required bending energies associated with making changes to a 2-dimensional object using the least amount of force. If an object is compared to itself, all lines would be parallel or perpendicular to all other lines, and all grid squares would be of equal size. These TPS grids are shown at 3x magnification to clarify changes. Parallel lines represent no change between objects, and all non-parallel lines represent a change in shape. Also, lines further apart represent a relative widening of that region relative to the rest of the structure, whereas lines that are closer together represent a relative shrinking of that region. Landmarks are as in Fig 2 and specimens are as described in Table 1.

**Fig 7:**
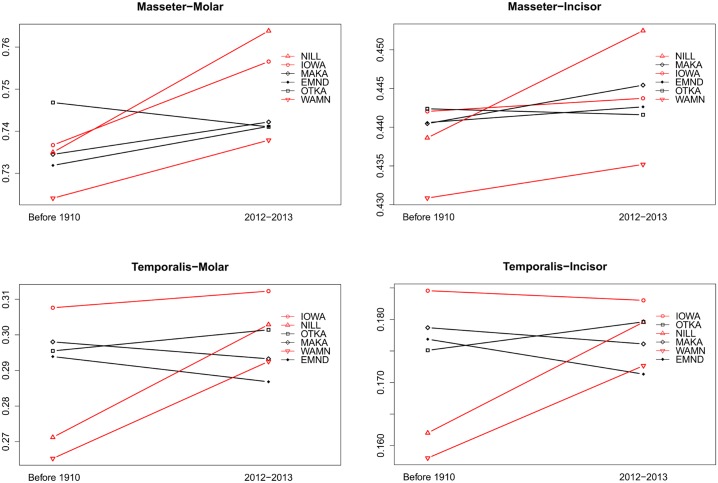
Biomechanical ratios of jaw morphology. Biomechanical ratios represent a measure of bite strength, and thus larger values represent greater bite strength at either the incisor or molars. Masseter length is the length from the condyle tip (jaw leverage) to the point of maximum curvature of the ventral side of the mandible. Temporalis length is the length from the condyle tip to the tip of the coronoid process. Incisor length in our study was the length from the condyle tip to the dorsal insertion point of the incisor (see methods for description of why the incisor was not included in the study). Molar length is the length between the condyle tip and the anterior insertion point of the first molar. These lengths are slight modifications from Anderson et al. [39]. Values in all graphs are ratios of lengths and are thus unitless. Site abbreviations and sample sizes are as in Table 1. Sites with greater than or equal to 75% corn-soybean cover in the landscape are depicted in red, while sites with less than or equal to 33% corn-soybean cover are depicted in black.

Thin plate spline deformation grids show that there were patterns of change in average mandible and maxilla morphology since 1910 (Fig 6). In general, the ramus and condyle had the most consistent expansion (both anteroposteriorly and dorsoventrally) in the mandible. The expansion in the ramus was more striking in the specimens from regions of high corn-soybean agriculture (IOWA, WAMN, NILL), while the condyle expanded in regions of high and low corn-soybean agriculture. The coronoid process is also rotated anteriorly in landscapes of high percentage corn-soybean, but maintains the historical posterior directionality in areas of low percentage corn-soybean agriculture. The upper jaw shows less distinction among sites. However, there appears to be a broadening (both anteroposteriorly and dorsoventrally) of the zygomatic plate or the portion of the maxilla lying directly above the molar row. The coronoid process, ramus, condyle, and zygomatic plate are all attachment regions for masticatory muscles, supporting a shift in diet from softer and smaller food items to a reliance on larger, harder food items. In general, biomechanical ratios increased between 1910 and 2012 (Fig 7), though overall changes were not significant (p > 0.05). These increases are generally more pronounced for specimens from high corn-soybean landscapes.

## Discussion

Our study was able to detect significant differences in the morphology of contemporary and historic specimens. We also found allometric heterochrony, the evolution of shape change with growth, in masticatory structures (Fig 4). The consistently different values for contemporary mandibles indicate that young and old adult mice have different morphologies than their historic counterparts. Lopez et al. [40] found similar results in two species of snakes that exhibited consistently different morphologies across sizes, likely associated with different diets. We also found a significant shift in the rate of morphological change associated with size for upper jaws (Fig 4). Magnhagen and Heibo [41] found similar changes in the rate of development in pike, again associated with differing diets.

In order to better understand the significant changes we found in shape, we compare our results to nongenetic, plastic responses to food hardness which indicate what types of changes in morphology might be associated with harder to process foods. The plastic changes seen in food hardness studies of mice are typically an overall broadening of muscle attachment points with exposure to hard food types [39,26]. For example, Myers et al. [26] found that *Peromyscus maniculatus* raised on a hard diet in the laboratory had a broader zygomatic plate, similar to our study (Fig 6). Renaud and Auffray [22] examined plasticity in domestic mouse (*Mus musculus domesticus*) mandibles, by raising mice on foods of different consistencies. The major morphological changes they described are movement in the incisor alveolus, uplift of the molar region, and change in the angular process. The changes they detected illustrate a similar broadening of the posterior half of the mandible, as in our study (Fig 6).

Our study found a significant difference between historical and contemporary specimens despite variation in the time of year when trapping occurred, as well as the fact that trapping occurred historically over several years and contemporary specimens were collected over two years. In addition, since *P*. *m*. *bairdii* is omnivorous, and utilize seeds and insects throughout the spring and summer months, their diets would have varied seasonally [12,42]. This variability, were it only to affect plastic developmental responses, would actually obscure differences between our historical and modern samples by increasing the variation in the shape of modern and historical specimens, part of which may explain the inconsistency in the condyle expansion being found in both high and low intensity locations, but not in all locations, as it has been previously found to be quite plastic [22]. Despite these factors, we found significant overall changes, which were best visualized by changes that match skeletal structures involved in masticatory muscle attachment, and by extension are associated with increased bite force [43] (Fig 6). For example, the enlarged maxillary zygomatic process, maxilla, and coronoid process are attachment points for the superficial and deep masseter muscles as well as the zygomatico-mandibularis muscle [43]. Williams et al. confirmed that jaw-muscle anatomy was predictive of bite force in small rodents [19]. They found that bite force is optimized at 40–50% gape, and that specialized skeletal morphology interacts with musculature to promote strong bites. Although we could not directly assess the influence of new morphologies on gape, we can ascertain that muscle attachments have changed. The enlargements of these muscle attachment regions may provide additional strength at all gapes, overcoming a lower bite force at large gapes. Also supporting this is the qualitative increase in biomechanical ratios, which represent an increased bite force, especially in areas of high corn-soybean landscapes.

Rapid morphological changes similar to those found in our study have been recorded in other natural populations that were also experiencing intense human-caused natural selection pressure [44]. In this study, the morphological adaptations likely contribute to the persistence of *P*. *maniculatus* in this extremely human-dominated landscape. Additionally, this morphological change may also have supported a shift in the species’ role in this ecosystem. Specifically, *P*. *m*. *bairdii* now consumes large quantities of weed seeds and waste grain, potentially reducing the amount of chemicals needed for control of weeds [15]. This is a major shift, as the species was just one of many small mammals in prairie ecosystems, consuming a wide variety of food. In order to protect new and valuable services from species such as *P*. *m*. *bairdii*, we should take careful note of other phenotypic changes that affect the ecosystem services being provided. Our lab has found that *P*. *m*. *bairdii* have adapted to this novel ecosystem in multiple ways, including the morphological changes in this study, a behavioral awareness of risk associated with barren landscapes and light soils, and discrimination between short- and long-term seed bank exotic plant species for consumption and caching [42]. All of these adaptations have allowed a common mouse to become an economically and environmentally significant species of the United States Corn Belt.
